# The effects of genotypes and media composition on callogenesis, regeneration and cell suspension culture of chamomile (*Matricaria chamomilla* L.)

**DOI:** 10.7717/peerj.11464

**Published:** 2021-05-24

**Authors:** Aqeel Ahmad, Muhammad Tahir ul Qamar, Almeera Shoukat, Mehtab Muhammad Aslam, Mohsin Tariq, Mansor Hakiman, Faiz Ahmad Joyia

**Affiliations:** 1 Faculty of Life Sciences, Department of Biotechnology, University of Central Punjab, Lahore, Pakistan; 2 State Key Laboratory for Conservation and Utilization of Subtropical Agro-Bio resources, College of Life Sciences and Technology, Guangxi University, Nanning, China; 3 Center for Plant Water-Use and Nutrition Regulation, College of Life Sciences, Joint International Research Laboratory of Water and Nutrient in Cops, Fujian Agriculture and Forestry University, Fuzhou, China; 4 Department of Bioinformatics and Biotechnology, Government College University, Faisalabad, Faisalabad, Pakistan; 5 Department of Crop Science, Faculty of Agriculture, Universiti Putra Malaysia, Serdang, Malaysia; 6 Laboratory of Sustainable Resources Management, Institute of Tropical Forestry and Forest Products, Universiti Putra Malaysia, Serdang, Malaysia; 7 Centre of Agricultural Biochemistry and Biotechnology (CABB), University of Agriculture Faisalabad, Faisalabad, Pakistan

**Keywords:** Callus induction, Cell suspension, Chamomile, Direct and indirect regeneration

## Abstract

**Background:**

Chamomile is an important herb being used widely for medicinal purposes. Its multitherapeutic, cosmetic, and nutritional values have been established through years of traditional and scientific use and research. Increased use of medicinal plants necessitates rational use as well as sustainable production of such genetic resources. Plant in vitro micro-propagation poses unique opportunities for sustainable production of medicinal herbs, their regrowth and conservation. The present study aimed to investigate the effects of different explants, plant growth regulators (PGRs) combinations and media type on callogenesis, in vitro regeneration and cell suspension of six chamomile genotypes to enhance its sustainable production.

**Methods:**

The shoot, lateral sprout, and leaf derived explants of six chamomile genotypes including Isfahan, Shiraz, Kazeron, Goral, Sharokashari and Presso were used for direct and indirect regeneration. For indirect regeneration various doses of NAA and kinetin were used to induce calli which were cultured on MS media containing PGRs for direct and indirect regeneration. Later, cell suspension was established and morphological characterization of CrO_3_ stained cells was carried out using microscopy.

**Results and Discussion:**

Our findings revealed that the highest callus percentage and callus volume were observed from lateral sprouts and shoots of genotype Isfahan on MS medium containing 1 mg/L NAA and 1 mg/L kinetin. The in vitro regeneration was found to be genotype dependent while 77% and 77.5% was the highest percentage for indirect and direct regeneration, respectively. Additionally, the maximum shoot number (two shoots/explant) and shoot length (2.22 cm) were also observed in Isfahan genotype. Cell suspension culture showed the highest fresh weight (18.59 g) and dry weight (1.707 g) with 0.75 g inoculum of the callus derived from lateral sprouts cultured on MS medium. Microscopy of CrO_3_ stained cells was carried on each 3rd day for 27 days that revealed larger and spongier cells in the early days as compared to final days when the cell number was greater but cell size was smaller.

**Conclusion:**

The callogenesis, organogenesis, and cell suspension culture of chamomile may be genotype dependent. Hence, optimization of media ingredients and culture conditions is of utmost importance for devising tissue culture based conservation strategy of any chamomile genotype and secondary metabolite production.

## Introduction

Chamomile (*Matricaria chamomilla* L.) is an important medicinal plant belonging to the Asteraceae family used in pharmaceuticals, hygiene, cosmetics and food industries (Park et al., 2017). Chamomile is known to exist in more than 120 constituents (McKay & Blumberg, 2006), imparting anticancer (Shoeb et al., 2001a), analgesic, anti-convulsive, carminative, anti-seizure (Ghanavatifard, Mohtadi & Masoumiasl, 2018), anti-inflammatory, antibacterial, sedative, immune booster, anti-allergic, anti-inflammatory, antiseptic and smooth muscle relaxant properties (Sotiropoulou, Megremi & Tarantilis, 2020). Increased use of herbal medicines has threatened the survival of a large number of medicinally important plant species (Abbas et al., 2018; Mumtaz et al., 2017; Thorat et al., 2017). However, development of sustainable crop production technologies as well as conservation strategies is inevitable (Abbas et al., 2020; Tahir ul Qamar & Khan, 2017). Plant in vitro micro-propagation presents unique opportunities for sustainable production of medicinal herbs, their rehabilitation and conservation, imparting of somaclonal diversity to increase the production of essential oils and other valuable secondary metabolites (Fay, 1992). Micropropagation, root and shoot growth derived from in vitro culture is severely affected by numerous factors such as species, genotype, culture medium, salts, organic matter, PGRs and environmental conditions. The type and concentration of auxin and cytokinin are key determinant factors affecting in vitro callogenesis and regeneration. Auxin to cytokinin ratio is the most important factor in this regard. Embryogenic calli are capable of regenerating through embryogenic and organogenic processes, while its regeneration ability is lower in non-embryogenic calli (Bright & Jones, 2012; Nic-Can et al., 2015). Transgenic plant production, propagation, and other tissue culture-dependent activities will not be effective without in vitro plant regeneration (Ebrahimie et al., 2006b). Plant regeneration is merely possible in two ways: either direct or indirect regeneration. Different genotypes have different regeneration abilities and may require specific explants for regeneration. Typically, explants with active mitotic cells are considered more appropriate for callus production (Ebrahimie et al., 2006b). Cell suspension culture is a collection of rapidly growing cells, which is created by transferring pieces of undifferentiated masses (calli) to a liquid culture medium. The purpose of cell suspension culture in plants is to achieve uniform and fast-growing cells especially to produce secondary metabolites (Grzelak, Janiszowska & culture, 2002; Phillips, Hubstenberger & Hansen, 1995). Previous studies on *Calendula officinalis* revealed that the highest callus induction rate was obtained by using 2,4-dichlorophenoxyacetic acid (2,4-D), Kinetin (Kin) and Indole acetic acid (IAA). Moreover, their results on the cell suspension suggested that the highest fresh and dry weight of the cells was observed on MS medium augmented with 2,4-D and kinetin under dark conditions (Saito & Nakano, 2002). In another study, the in vitro regeneration of *Hostaseiboldiana* from cell culture was reported and the highest amount of fresh and dry weight of cells was observed in liquid MS medium containing 0.5 mg/L of picloram and inoculum of 200 mg callus. The advantages of cell suspensions are short cell cycle, lack of dependence on environmental conditions such as climate, soil quality, growing season, day length and high biosafety (Cisneros-Torres et al., 2020; Xu, Ge & Dolan, 2011; Yue et al., 2016). Very limited reports are available on tissue culture and micro propagation of chamomile. (Saito & Nakano, 2002) reported that the highest number and length of shoots were obtained from nodal fragments of chamomile on MS medium augmented with 1 mg/L 2,4-D. Delamare et al. (2000) revealed that the formation of adventitious sprouts and shoots of chamomile under in vitro conditions were induced on MS medium augmented with 1 mg/L benzylaminopurine (BAP) and 0.18 mg/L NAA (naphthaleneacetic acid). Therefore, present study was designed to present an improved protocol for callus induction, regeneration and cell suspension culture of six chamomile genotypes (Isfahan, Shiraz, Kazeron, Guaral, Sharokashari, Presso) with the aim of enhancing its conservation and improving secondary metabolite production via cell suspension culture.

## Materials & Methods

### Plant material and explants preparation

Seeds of six genotypes including Isfahan, Shiraz, Kazeron, Goral (2n = 4x = 36), Sharokashari (2n = 2x = 18), and Presso (2n = 2x = 18) used in this study were commercially purchased from Sky Seeds Lahore, Pakistan (https://www.skyseeds.pk/). After germination in plastic pots, seedlings with 10–15 cm height were disinfected in 1% sodium hypochlorite solution for 60 min and then in 70% ethanol for 10 min. The seedlings were rinsed four times with sterile distilled water for 4 min each time. Shoot, lateral sprout, and leaves explants were cut into 0.5 cm and placed on MS medium. This study was divided into three main sections: callogenesis, regeneration, and cell suspension.

### Callogenesis optimization

To determine the best callogenesis medium, a factorial experiment based on a completely randomized design with three treatment factors including NAA (0, 1, 2, and 4 mg/L), and kinetin (0, 0.5, 1 and 2 mg/L) and explant (shoot, lateral sprout, and leaf) with three replications, was performed. The container was transferred to dark room for two weeks and provided a photoperiod 16/8 (Day/Night), 26 ± 1 °C for callogenesis. The best PGRs combination was selected based on the desired traits and used in subsequent experiments.

### Callogenesis from different chamomile genotypes

After optimization of the callogenesis medium, different explants of chamomile genotypes were studied for callus-related traits such as callogenesis percentage and callus volume which was measured by the Hooker scale (Hooker & Nabors, 1977). This experiment was conducted as a factorial experiment based on a completely randomized design with two factors including genotypes (Goral, Sharokashari, Presso, Isfahan, Shiraz, Kazeron) and explants (shoot, lateral sprout, leaf) with three replications. Callogenesis characteristics were measured four weeks after cultivation.

### In vitro regeneration

#### Indirect regeneration

Calli with compact and grainy texture having green and yellow colors were selected from three explants (shoots, lateral sprouts and leaf explants) and transferred to regeneration media. For shoot regeneration, two separate factorial experiments based on a completely randomized design were designed using the above-mentioned calli. The studied factors in the first experiment included investigating the individual effect of PGRs (Kin 0.5, 1, 2, 4 mg/L and NAA 0, 0.5 mg/L on calli induction). While the second experiment investigated the impact of different PGRs combinations (BAP 0.5, 1, 2, 4 mg/L and NAA 0, 0.5 mg/L) on callogenesis and subsequent on direct regeneration of calli by using same explants.

#### Direct regeneration

After selection of the best PGRs composition for the production of high vigor shoots at the stage of callus optimization, a direct regeneration experiment was conducted to investigate the ability of direct organogenesis in studied genotypes. The factorial experiment in a completely randomized design was conducted in triplicate with two factors including six genotypes and three explants.

### Cell suspension culture

The cell suspension culture was prepared using a medium containing the best PGRs composition without adding agar. This study was conducted as a factorial experiment based on the completely randomized design with three factors including explants (calli derived from the shoots, lateral sprouts, and leaves), the mass of inoculum (0.5, 0.75, 1 g/L) and culture media (MS and B5). The culture medium was divided into 250 mL container and each included 50 mL liquid suspension. After that from prepared culture, 0.5 to 0.75 g was transferred to 250 mg container. Then the container was closed and samples were kept on orbital shaker at 120 rpm for first 24 h and then at 100 rpm for next 24 h and finally kept at 90 rpm and 26 ± 1 °C (Abdi, Mehrabi & Zarei, 2014). Overall cell biomass was calculated by measuring the fresh and dry weight of cells. Morphological characteristics of the cells in cell suspension were also studied through microscopy. Fresh and dry weights of the cells were measured after 24 days of the culture initiation by using filter paper and vacuum pump. The chromium trioxide staining method was used to differentiate cells from each other (Wilson & Street, 1975). Cell counting was performed at the intervals of every 3 days for up to the 27th day of culture as follows. First, under sterile conditions, a culture medium containing one mL of the cultured cells was mixed with four mL of chromium trioxide solution (12%), placed in a Bain Marie for 5 to 20 min at 60−70 °C. Then, the mixture was stirred for five seconds using a vortex machine at room temperature. The number of cells was counted using a × 10 optical microscope. Two µl of the mixture was placed on a glass slide for microscopy to check the cell size and shape and to calculatethe viability percentage.

### Statistical analysis

All experiments were carried out in a factorial experiment based on a completely randomized design. Statistical analysis was performed using SAS (Statistical Analysis System) software (SAS Institute, Cary, NC). The ANOVA (analysis of variances) and LSD (Least significant difference) test were perfomed for statistical analysis.

## Results

### Effect of PGRs combinations on callogenesis attributes

**Figure 1:**
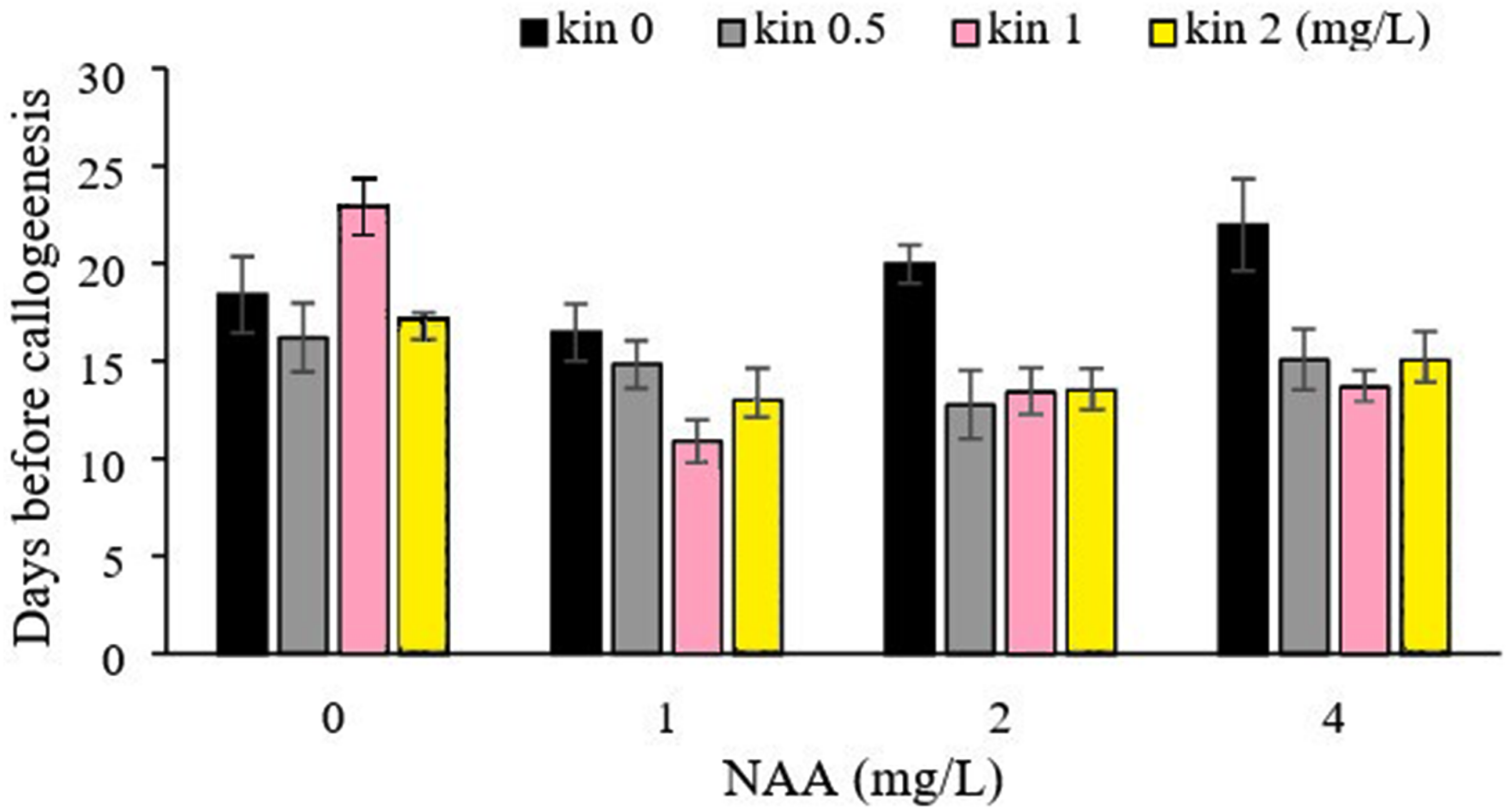
Effect of different concentrations of NAA and kinetin on the beginning of callogenesis. The mean comparison of NAA and kinetin combination (1 mg/L NAA + 1 mg/L kinetin) led the lowest number of days (10.89) required for callogenesis.

Highest callus induction frequency of chamomile was recorded on medium containing combinations of NAA and kinetin compared to 2, 4-D, and kinetin combinations reported previously (Bakhtiar et al., 2014). Hence, NAA and kinetin combination was selected to perform further subsequent experiments. Statistical analysis indicated that the triple interaction of NAA, kinetin, and explants treatments was significantly different for all evaluated traits except starting days before callogenesis (Table 1). It is worth mention that the statistical variance of the NAA/kinetin and kinetin/explant interaction showed significance for callogenesis. The mean comparison of NAA and kinetin combination (1 mg/L NAA + 1 mg/L kinetin) led the lowest number of days (10.89) required for callogenesis (Fig. 1).

**Table 1:** Analysis of variance of the effect of NAA, Kin, and explant on the measured characters.

S.O.V.	df	%Days before callogenesis	%Callogenesis	Callogenesis Volume (cm^3^)	%Embryogenesis	%Rooting
NAA	3	164.44^**^	6342.9^**^	137.3^**^	2154^**^	427.4^ns^
KIN	3	149.54^**^	4558.9^**^	103.6^**^	5394.5^**^	8397.3^**^
Explant	2	133.53^**^	570^ns^	3.77^ns^	931.3^*^	1931^*^
NAA*Kin	9	52.05^**^	1456.3^**^	13.41^**^	1111.3^**^	1570^**^
NAA* Explant	6	53.10^*^	924.5^**^	21.03^**^	517.5^ns^	1193^ns^
Kin* Explant	6	15/86^ns^	138.5^ns^	7.25^ns^	379.7^ns^	851.8^ns^
NAA*Kin* Explant	18	21/85^ns^	448.9^*^	9.76^**^	968.5^**^	271.6^ns^
Error	96	18/56^ns^	260	4.5	263.9	570.02

**Notes.**

ns,*,** non-significant, significant at the 0.05 and 0.01 probability level, respectively.

**Figure 2:**
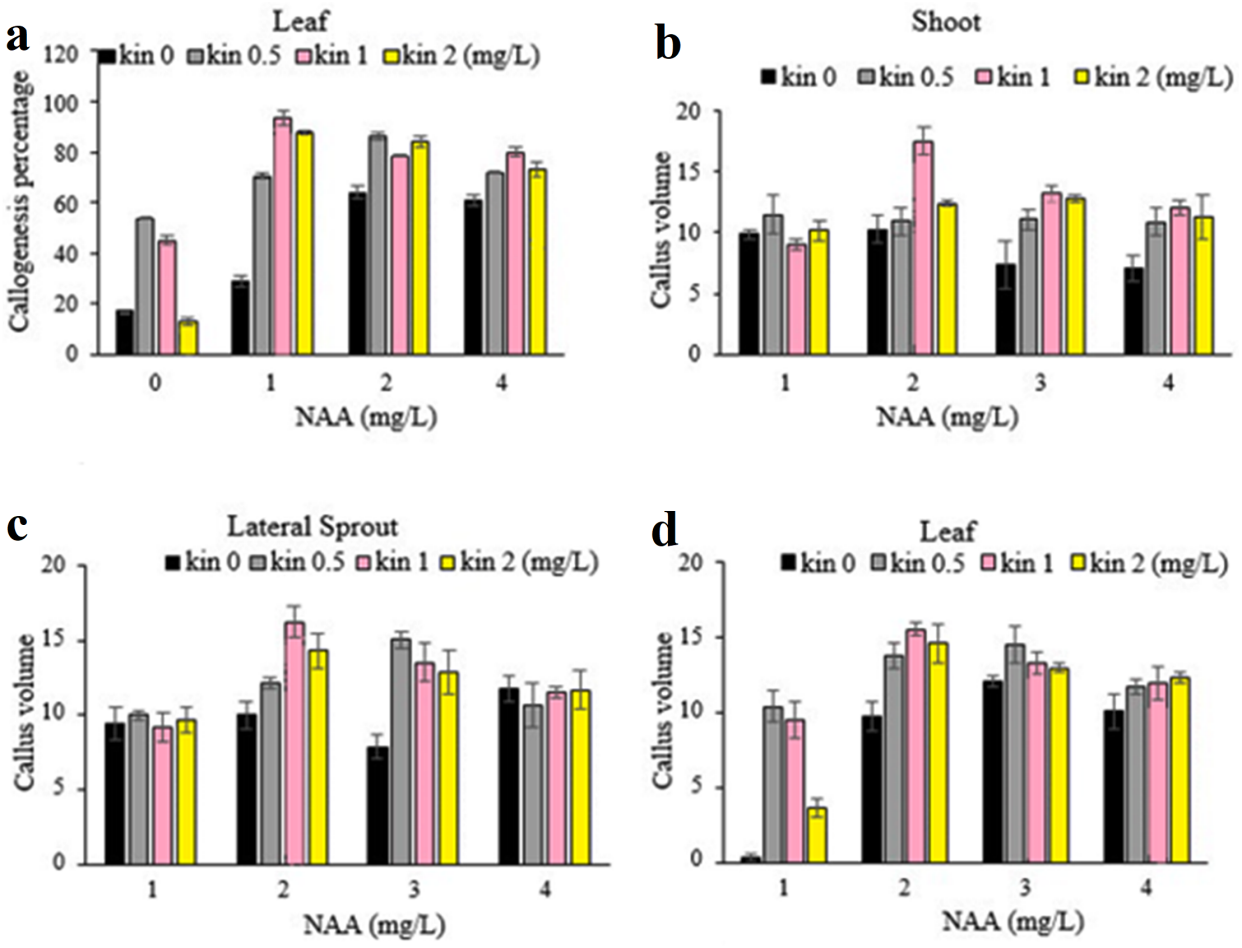
(A-D) Effect of different concentrations of NAA and kinetin on the callogenesis percentage and callus volume (cm^3^) among different explants.

For callogenesis potential, the highest percentage of callogenesis with a mean value of 93.26% was obtained on a medium containing 2 mg/L NAA and 1 mg/L kinetin (Fig. 2). Therefore, this PGRs combination was selected to perform further subsequent experiments. Talking about callus volume, shoot explants produced the maximum callus volume (17.49 cm^3^) on medium containing 1 mg/L NAA and 1 mg/L kinetin while the highest callus volume in lateral sprout (16.20 cm^3^) and leaf explants (15.52 cm^3^) was also observed on the same PGRs combination.

**Figure 3:**
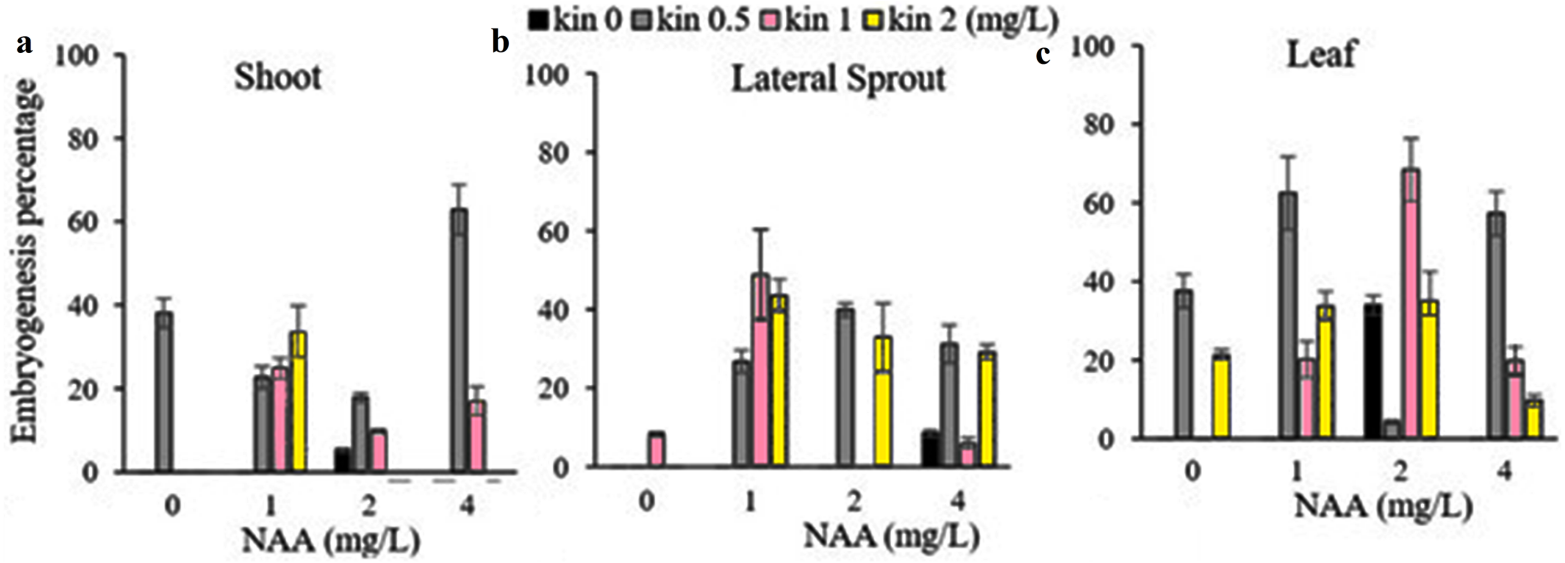
(A-C) Effect of various concentrations of NAA and kinetin and different explants on embryogenesis percentage efficiency of the shoot, lateral sprout and leaf.

**Figure 4:**
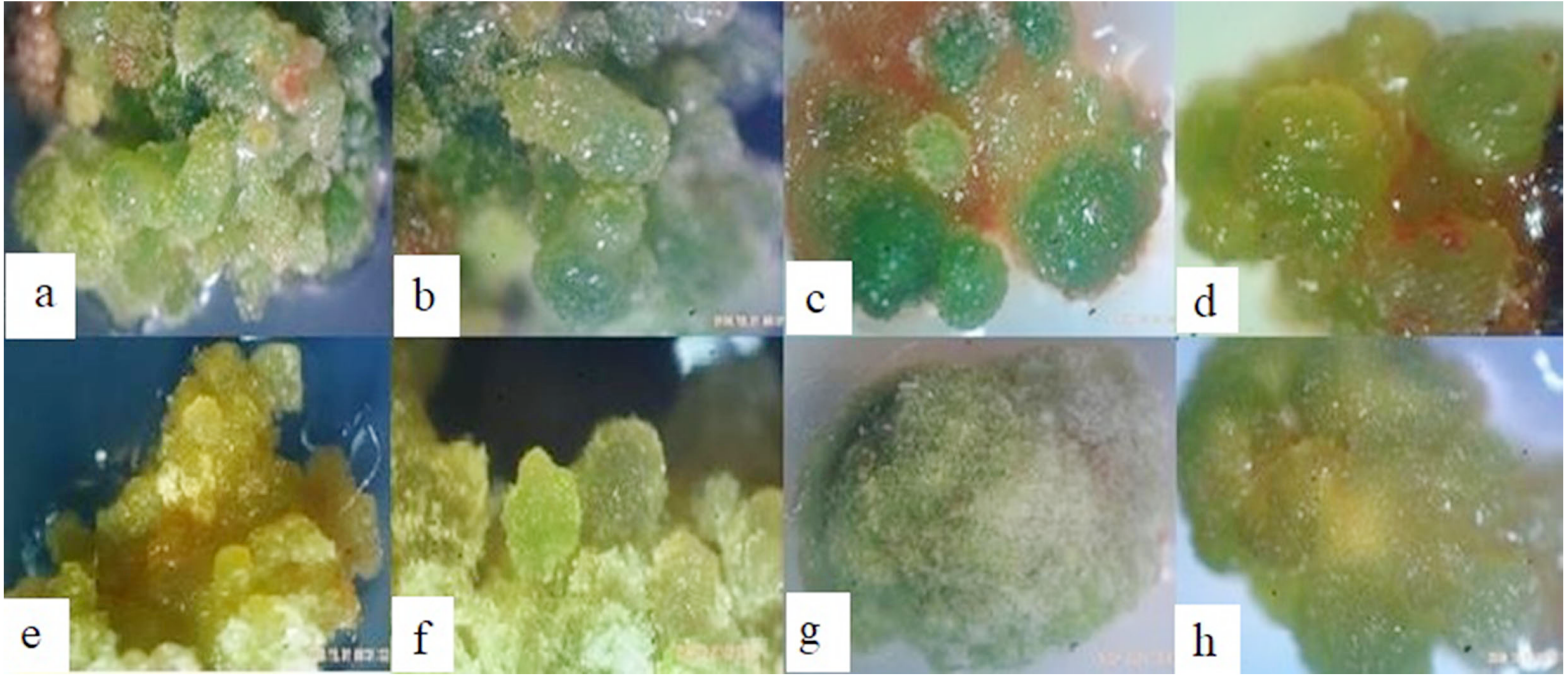
The physical appearance of embryonic callus induction from different explants. (A–B) Leaf explants grown on medium containing 2 mg/L NAA and 1 mg/L Kin, (C–D) shoot explants grown on medium containing 4 mg/l NAA and 0.5 mg/L Kin, (E-F) lateral sprouts on medium containing 1 mg/L NAA + 1 mg/L of kin, (G–H) non embryogenic callus on PGRs-free medium used as control.

Our findings revealed that leaf explants produced the highest embryogenesis percentage (68.37%) on medium supplemented with 2 mg /L NAA and 1 mg/L kinetin; while the maximum embryogenic callus (62.83%) derived from shoot explant and lateral sprout explants (48.86%) were observed on medium containing 4 mg/L NAA + 0.5 mg /L kinetin, and 1 mg/L NAA + 1 mg/L kinetin respectively (Fig. 3). The above-mentioned PGRs treatments produced variable %age of embryogenic calli, while PGR free medium resulted in non-embryogenic calli formation (Fig. 4).

### Genotypic effect on callogenesis attributes

Our findings revealed a highly genotype-dependent response of chamomile to plant regeneration ability. Callus induction from different explants started approximately 10–13 days after culture initiation. The highest callus volume (16.16 cm^3^) and callogenesis percentage (78.83%) were observed in the Isfahan genotype (Table 2). Mean comparison of callogenesis and callus volume depicted the highest volume of callus (14.9 cm^3^), and the highest percentage of callogenesis (66.8%) was obtained from lateral sprout explants. Although all explants (shoots, lateral sprout, and leaves) were able to produce calli, the volume of calli was genotype-dependent. Therefore, it seems that callogenesis induction is highly genotype-dependent in chamomile, thus genotypes with high callogenesis ability should be screened to produce calli for cell suspensions and subsequent extraction of secondary metabolites. In the current study, the highest rate of callogenesis was obtained in Isfahan, followed by Kazeron, Goral, Sharokashari, Shiraz, and Presso genotypes. The leaf callogenesis percentage of leaf was higher than the lateral sprout and shoot, and it can be attributed to the difference in the callogenesis potential of different explants, the phytochemical composition of the explants, and the concentration/combination of endogenous PGRs and above all the genotype used (Mansouri, Fadavi & Mortazavian, 2015). Previous studies also showed variable callogenesis response to different explants and genotypes, indicated that the overall callogenesis ability is a product of genotype/explant interaction (Shoeb et al., 2001b).

**Table 2:** Mean comparison of callogenesis and callus volume of different explants on chamomile genotypes.

Factor	Levels	Callogenesis%	Callus volume (cm^3^)
Genotype	Isfahan	78.83 a ±5.40	16.16 a ± 0.87
	Kazeron	67.42 ab ± 8.85	13.27 bc ± 0.7
	Goral	64.12 ab ± 6.64	14.66 ab ± 0.94
	Sharokashari	49.76 bc ± 5.40	13.60 bc ± 0.76
	Presso	43.48 c ± 6.50	11.87 c ± 0.81
	Shiraz	36.91 c ± 7.39	11.85 c ± 0.49
Explants	Leaf	66.98 a ± 5.80	11.72 b ± 0.46
	Shoot	50.40 b ± 4.21	14.39 a ± 0.7
	Lateral sprouts	54.88 ab ± 4.84	14.99 a ± 0.54

### In vitro regeneration

#### Indirect regeneration

**Figure 5:**
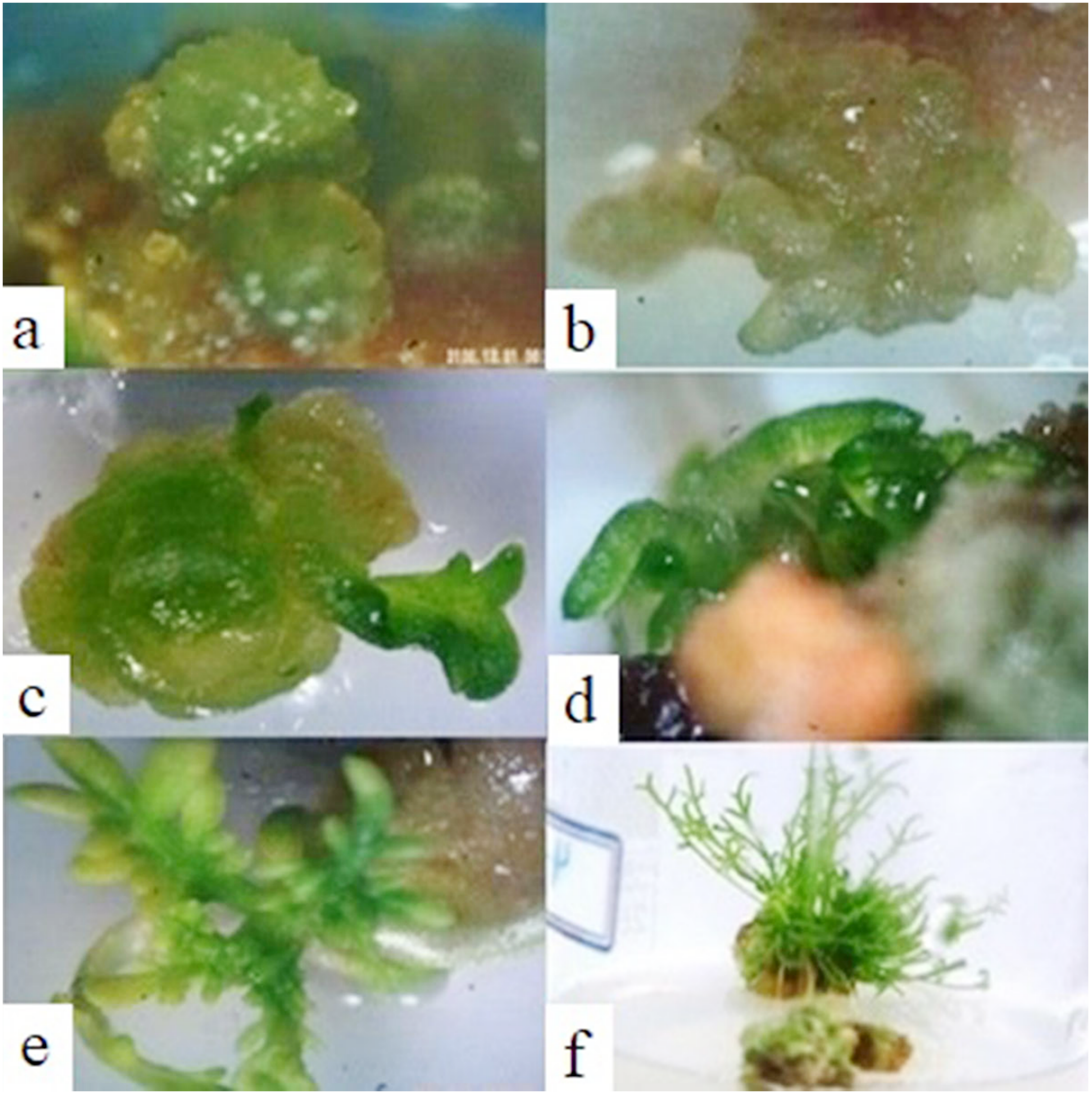
Indirect regeneration of the chamomile leaf-derived calli on a medium containing 0.5 mg/L of kinetin. (A–B) The appearance of primary nodes on the calli, (C) newly developed leaf formation, (D) Callus regeneration with the emergence of new shoots, (E–F) shoot proliferation.

Indirect regeneration response of calli induced by using different explants along with different PGRs combinations showed highly variable responses. It was observed that a medium containing only Kinetin is useful for indirect regeneration. Leaf-derived calli were successfully regenerated indirectly on the medium containing 0.5 mg/L of kinetin (Fig. 5). The cultured calli did not regenerate on other media and some of them underwent necrosis after one month of culturing. It indicated the inhibitory role of higher auxin levels in regeneration process of the same line (Ammirato & Steward, 1971).

**Figure 6:**
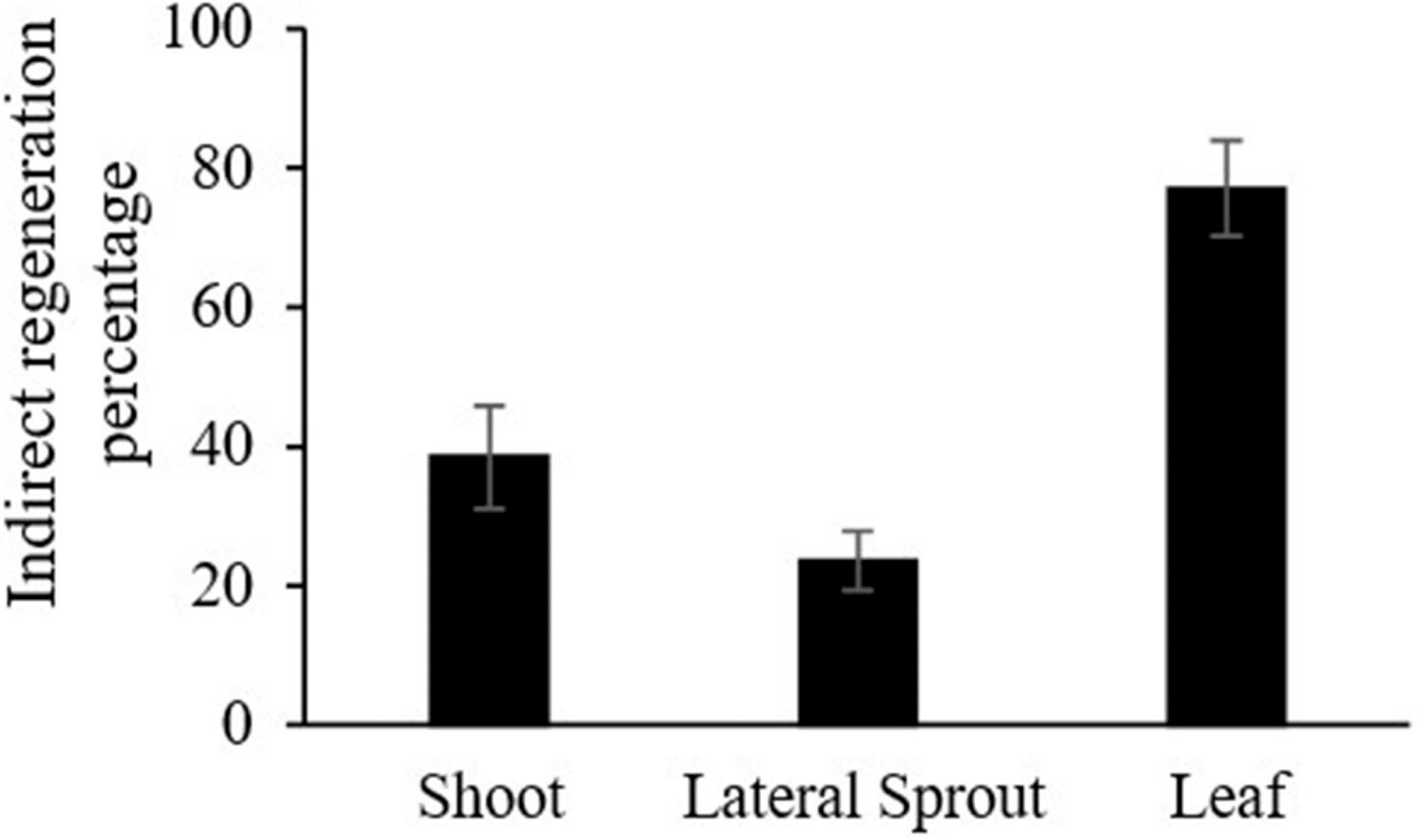
Comparison of the percentage of indirect regeneration in the calli derived from different explants.

The regeneration percentage of the calli derived from the shoot, leaf, and lateral sprouts was significantly different and the highest regeneration was recorded from leaf-derived callus (77%) (Fig. 6). Therefore, it can be said that shoot regeneration is highly dependent on the explants and the endogenous PGRs. However, the external application of auxin and cytokinin is needed for the induction of adventitious sprouts and their differentiation into shoots. Previous studies also revealed a higher regeneration ability of the leaf-based explants than those of the hypocotyls and roots (Mujib & Šamaj, 2006).

### Direct shoot regeneration and root induction

**Figure 7:**
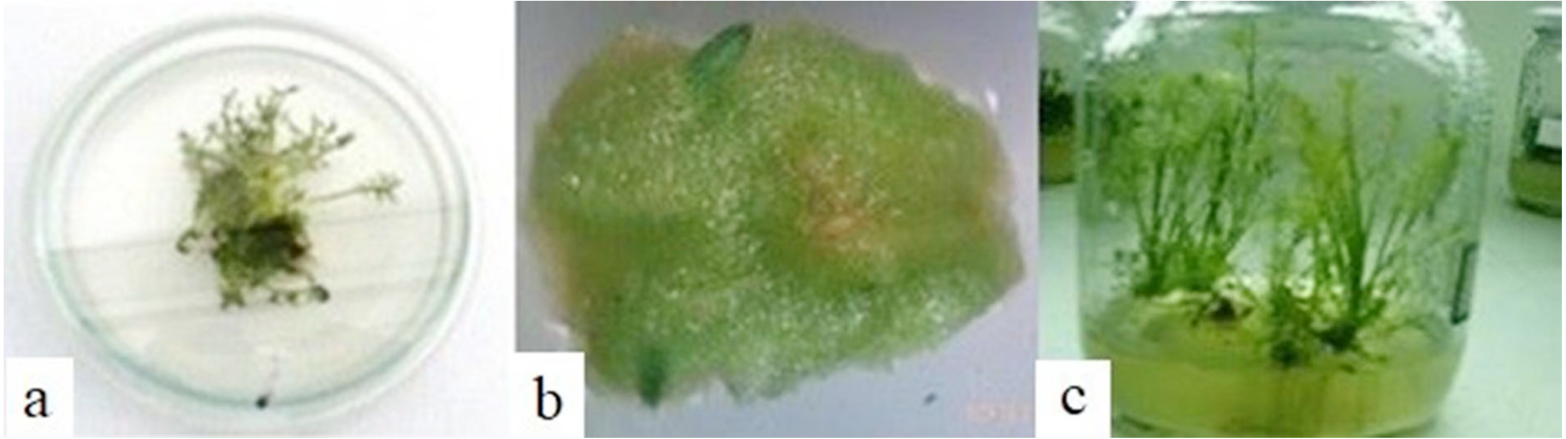
Direct regeneration of chamomile. (A) Shoot regeneration from the lateral sprout, (B) structures similar to the shoot obtained from Presso leaf explant, (C) shoot proliferation and root induction.

Direct shoot regeneration induced from lateral sprouts was better as compared to other explants among all genotypes. Leaf explants did not show any shoot regeneration (Fig. 7). Therefore, the data derived from the shoot and lateral sprouts were further subjected to analysis which showed a significant effect of genotype and different explants on shoot number and length. The highest number of shoots/explant (2) and the maximum shoot length were observed in the Isfahan genotype using the lateral sprouts (Table 3). Contrastingly, the Goral genotype and the lateral sprout explant produced the highest root number 8.97 and 5.53, respectively. Moreover, Goral genotype had the highest root length (6.22 cm), while different explants showed no differences for this attribute. The highest regeneration percentage was achieved in the Isfahan genotype (77.5%). Among the explants, lateral sprouts produced the maximum (69.48%) regeneration percentage (Table 3). Collectively, the results of the present study confirmed the importance of the genotype, explants, and their mutual interaction of chamomile on the direct regeneration method.

**Table 3:** Mean comparison of regeneration related traits in different chamomile genotypes and explants.

**Factor A**	**Factor B**	**No. of shoots**	**Shoot length (cm)**	**No. of roots**	**Root length (cm)**	**Shoot regeneration%**
Genotype	Goral	1.26 b ± 0.16	1.86 ab ±0.20	8.97 a ± 1.21	6.02 a ± 1.13	56.17 ab ± 9.82
	Sharokashari	0.84 b ± 0.15	1.05 c ± 0.22	2.68 b ± 1.28	1.05 b ± 0.60	37.08 bcd ± 8.17
	Isfahan	1.77 a ± 0.21	2.22 a ± 0.16	2.14 b ± 0.90	0.84 b ± 0.30	77.50 a ± 9.04
	Kazeron	0.83 b ± 0.17	1.08 c ± 0.35	1.42 b ± .66	0.58 b ± 0.28	31.79 cd ± 14.74
	Shiraz	1.17 b ± 0.08	1.80 ab ±0.24	2.19 b ± 1.34	0.58 b ± 0.27	53.54 bc ± 12.12
	Presso	0.88 b ± 0.30	1.40 bc ±0.45	1.63 b ± 0.98	1.42 b ±.05	25 d ± 11.11
Explant	Shoot	0.75 b ± 0.12	0.97 b ±0.18	1.98 b ± 0.76	1.27 a ± 0.27	19.25 b ± 3.67
	Lateral sprout	1.42 a ± 0.1	2.02 a ± 0.12	4.53 a ± 0.82	2.37 a ± 0.61	69.48 a ± 5.15

### Cell suspension culture

**Figure 8:**
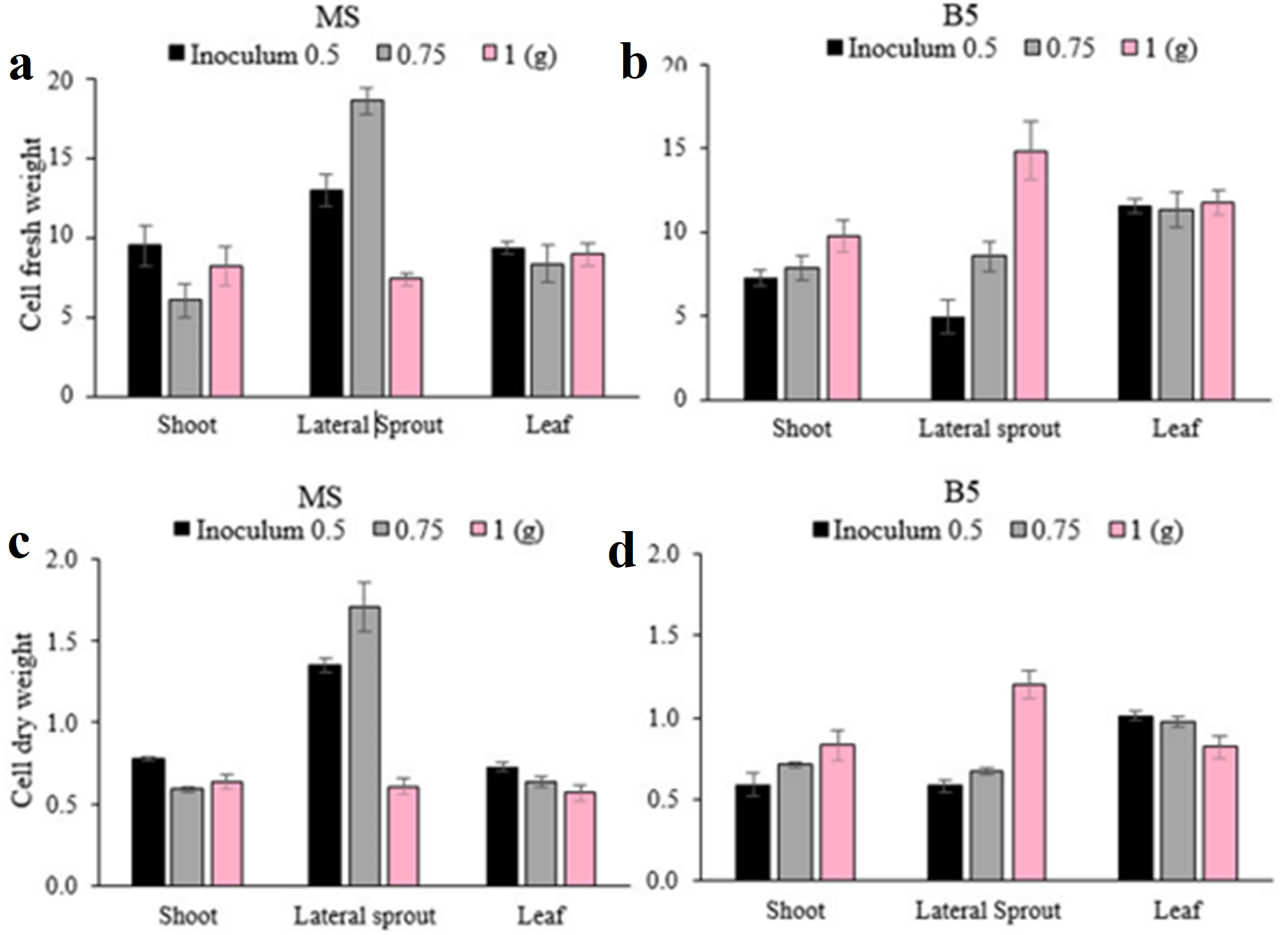
(A-D) Mean comparison of cell fresh and dry weights for the studied factors in cell suspension culture.

The most important factors for improving cell growth in cell suspensions are the amount of inoculum and the culture medium (Kim et al., 2001; Magiatis et al., 2001). Cell suspension culture showed that the highest cell fresh weight (18.59 g) and dry weight (1.707 g) were obtained using 0.75 g of inoculum of the callus derived from lateral sprouts on MS medium (Fig. 8). The highest cell fresh weight (14.84 g) and dry weight (1.2 g) were obtained using 1 g of inoculum from the lateral sprout-derived callus growing on the B5 medium. This highlights the importance of callus origin and medium is establishing successful suspension culture in chamomile.

**Figure 9:**
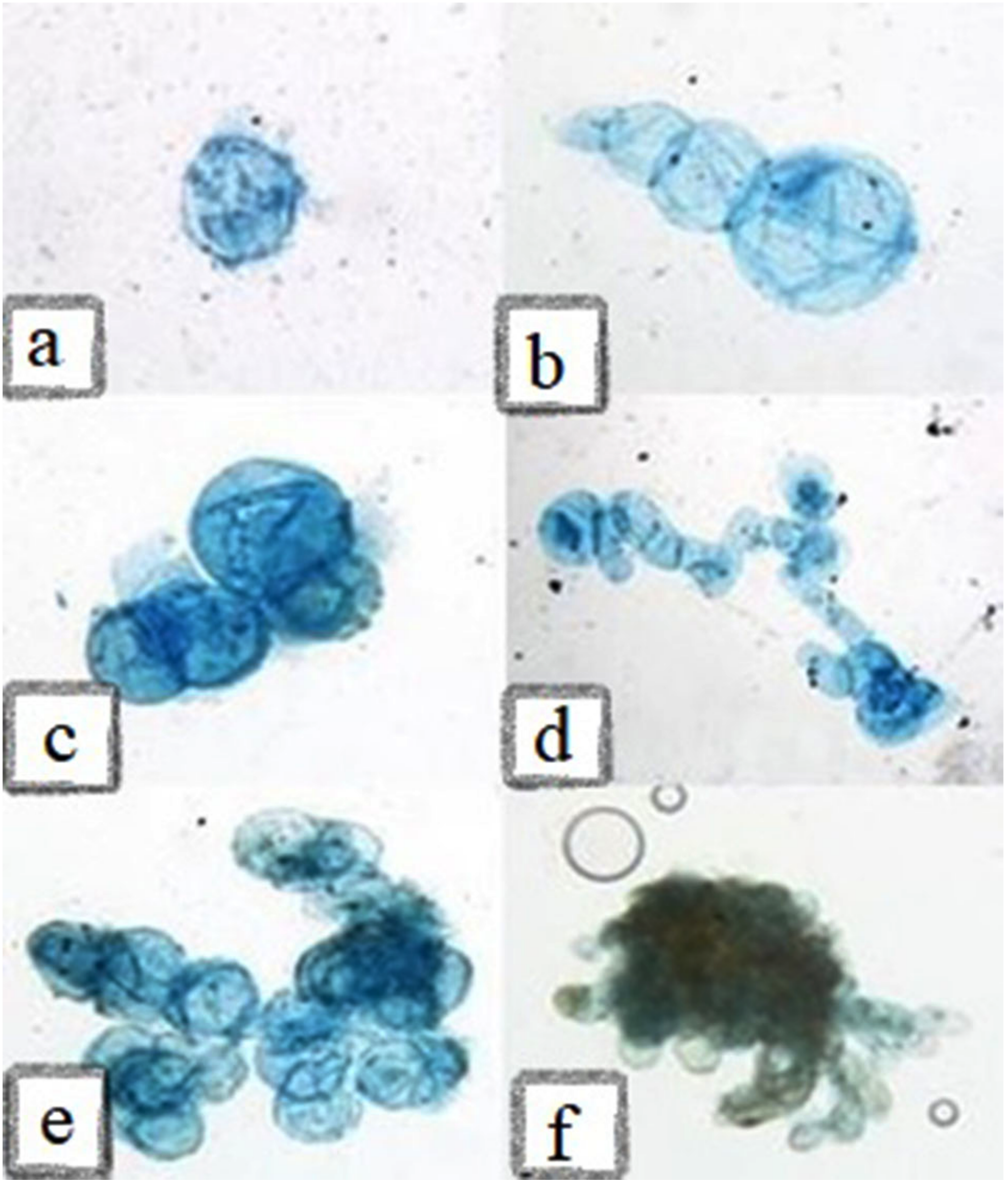
Microscopic illustration of staining cells with Evans Blue (0.1%). (A) Globular shaped cells with large size, (B) three single interconnected cells, (C-D) starting of cell masses in cell suspension, (E) cell masses with same-sized cells in the presence of 2-4-D, (F) cell masses with elongated cells in the presence of NAA.

The morphological characteristics of cells in suspension cultures were evaluated using microscopy. Cells in the suspension culture were often clustered and less likely to be seen in single and isolated cells (Fig. 9). In the present study, the cell masses in the presence of 2, 4-D formed were of equal size, while in the presence of NAA, their morphology changed to a string of beads. In general, the morphology and shape of the cells in suspension culture may vary based on the origin of inoculum cells. These results also showed that the cell shape in the early days of culture was larger and spongier than the middle days, while in the final days, the number of cells was higher, but their size was smaller. It has been reported that the cell size and shape in the suspension culture are affected by the type of auxin (Grzelak, Janiszowska & culture, 2002; Smolenskaya et al., 2007).

## Discussion

Chamomile is considered as recalcitrant for successful in vitro regeneration and callus induction. The regeneration and transformation protocol is limited and need to be extended to transform the locally adapted cultivars in many parts of the world.

In vitro regeneration and genetic transformation depend on PGRs, medium containing combinations of NAA and kinetin. Cell suspension culture in plants is to achieve a uniform and fast-growing cells mainly to produce secondary metabolites. The production of maximum callus in a minimum period is one of the important goals in tissue culture techniques, which not only saves time and cost but also prevents the emergence of possible somaclonal variations, which is reported here in comparison with other studies (Bakhtiar et al., 2014; Giménez et al., 2001). In this study, the highest percentage of callogenesis with a mean value of 93.26% was obtained on a medium containing 1 mg/L NAA and 1 mg/L kinetin. Previous studies indicated that different plant tissues vary in terms of callogenesis potential regarding the quality and quantity of their internal PGRs, and this ability was higher in leaf explants (Aghaei, Bahramnejad & Mozafari, 2013; Klemš et al., 1998).

The leaf explants produced the highest embryogenesis percentage (68.37%) on medium supplemented with 2 mg /L NAA and 1 mg/L kinetin. It has been reported that the sensitivity of cultured tissues and cells to external signaling PGRs can determine the totipotency potential of explants resulting in embryogenic/non-embryogenic calli (Aghaei, Bahramnejad & Mozafari, 2013). The level of endogenous PGRs is one of the key factors affecting the explants responses and embryogenesis ability of leaf explants compared to other explants (Mujib & Šamaj, 2006). The results presented here showed that embryogenesis ability is highly dependent on explants type and media composition, especially PGRs concentration. Similar studies have also stated that the type and concentration of PGRs, explants type, and their physiological stage are the most important factors affecting embryogenesis ability (Quiroz-Figueroa et al., 2002; Tawfik & Noga, 2001).

The present study revealed that the callogenesis induction is highly genotype-dependent in chamomile, thus genotypes with high callogenesis ability should be screened to produce calli for cell suspensions and subsequent extraction of secondary metabolites. In the current study, the highest rate of callogenesis was obtained in Isfahan, followed by Kazeron, Goral, Sharokashari, Shiraz, and Presso genotypes, and the callogenesis percentage of a leaf was higher than the lateral sprout and shoot. Previous studies also showed variable callogenesis response to different explants and genotypes, indicated that the overall callogenesis ability is a product of genotype/explant interaction (Shoeb et al., 2001b).

In indirect regeneration observed that a medium containing only kinetin is useful, and leaf-derived calli were successfully regenerated indirectly on the medium containing 0.5 mg/L of kinetin. The cultured calli did not regenerate on other media, indicating the inhibitory role of higher auxin levels in the regeneration process (Ammirato & Steward, 1971). These results can be explained by the fact that a high concentration of auxins in regeneration media results in the biosynthesis of hydrocarbons such as ethylene, which leads to senescence in plant cells and tissues. It is thought that removal of auxin from the medium is an effective approach at the beginning of the regeneration process, whereas the continued presence of high auxin concentrations in the medium prevents regeneration through photo-oxidative decomposition and the production of unwanted compounds (Lim, Ling & Hussein, 2009).

The regeneration percentage of the calli was found significantly different and recorded a high rate in leaf derived callus (77%). However, auxin and cytokinin are needed for the induction of adventitious sprouts and their differentiation into shoots. Previous studies also revealed a higher regeneration ability of the leaf-based explants than those of the hypocotyls and roots (Mujib & Šamaj, 2006). Direct shoot regeneration induced from lateral sprouts was better as compared to other explants among all genotypes which showed a significant effect of genotype and different explants on shoot number and length. The highest number of shoots/explant and the maximum shoot length was observed in the Isfahan genotype using the lateral sprouts. The Goral genotype produced the highest root number and root length. The highest regeneration percentage was achieved in the Isfahan genotype (77.5%). Since direct regeneration does not require time for callogenesis and successive subculturing. Moreover, it is not only cost-effective but also produces true-to-type plants by reducing the probability of somaclonal variations (Bakhtiar et al., 2014; Ebrahimie et al., 2006a; Pandey et al., 2015). The most important factors in improving cell growth in cell suspensions are to determine the amount of inoculum and the culture medium (Kim et al., 2001; Magiatis et al., 2001).

Cell suspension culture showed that highest fresh and dry weight using 0.75 g of inoculum of callus derived MS medium than 1 g of inoculum on B5 medium. It is reported that the highest cell fresh and dry weights were observed in the cell suspension of *Calendula officinalis* on MS medium containing 2,4-D, and kinetin under dark conditions (Grzelak, Janiszowska & culture, 2002). Contrarily, leaf was selected as desirable explant in MS medium containing 0.5 mg/L kinetin, 1 mg/L 2, 4-D, and 1 mg/L IAA in *Catharanthus roseus* (Valluri, 2009). Morphologically, the cell masses in the presence of 2,4-D formed were of equal size, while in the fact of NAA, their morphology changed and converted to a string of beads. It has been reported that the cell size and shape in the suspension culture are affected by the type of auxin (Grzelak, Janiszowska & culture, 2002; Smolenskaya et al., 2007). The quicker recovery of in vitro plants and extra-ordinary regeneration potential makes this variety an ideal candidate for future transformation-based studies. The regeneration conditions identified in this work will be useful for improving different genetic engineering approaches against various biotic and abiotic stresses as well as functional genomic studies.

## Conclusions

The callus optimization experiment showed that the combination of NAA and kinetin was more effective for the callogenesis of chamomile. The leaves, shoots, and lateral sprouts resulted the highest callogenesis percentage and callus volume on MS medium containing 1 mg/L NAA and 1 mg/L of kin. Callogenesis percentage and callus volume differed significantly among genotypes, where Isfahan genotype was found to be the best. Regeneration experiments indicated that the shoot number and shoot length were affected by genotype and explants type. The highest number of shoots and the maximum shoot length were observed in the Isfahan genotype using the lateral sprouts, while Goral genotype produced the highest root number and root length. The highest regeneration percentage was also obtained from the Isfahan genotype (77.5%). Based on the information gathered, Isfahan and Goral genotypes were the most appropriate genotypes. Cell suspension culture showed that the highest cell fresh and dry weights were obtained using 0.75 g of inoculum in the callus derived from lateral sprouts grown on MS medium. Microscopic evaluation of cell suspension revealed that in the presence of 2,4-D, the cell masses formed were of the same sizes. Taken together, it can be concluded that the response of different Chamomile explants and genotypes on different media types was different in terms of callogenesis, organogenesis, and cell suspension culture, and cell biomass production. Therefore, the selection of totipotent genotypes with more callogenesis ability, not only proved to be cost-effective but also applicable in cell suspension culture and extraction of secondary metabolites.

##  Supplemental Information

10.7717/peerj.11464/supp-1 Data S1 Raw Data

## Additional Information and Declarations

### Data Availability

The following information was supplied regarding data availability: The raw data associated with Tables 1– –3 and Figs. 1, 3, 6 and 8 are available as Supplemental Files.
